# Bonobo personality traits are heritable and associated with vasopressin receptor gene 1a variation

**DOI:** 10.1038/srep38193

**Published:** 2016-12-02

**Authors:** Nicky Staes, Alexander Weiss, Philippe Helsen, Marisa Korody, Marcel Eens, Jeroen M.G. Stevens

**Affiliations:** 1 Centre for Research and Conservation, Royal Zoological Society of Antwerp, Antwerp, Belgium; 2 Behavioural Ecology & Ecophysiology Group, Department of Biology, University of Antwerp, Antwerp, Belgium; 3 Center for Advanced Study of Human Paleobiology, Department of Anthropology, George Washington University, Washington DC, United States of America; 4 Department of Psychology, School of Philosophy, Psychology and Language Sciences, The University of Edinburgh, United Kingdom; 5 The Scottish Primate Research Group, United Kingdom; 6 San Diego Zoo Institute for Conservation Research, California, United States of America

## Abstract

Despite being closely related, bonobos and chimpanzees show remarkable behavioral differences, the proximate origins of which remain unknown. This study examined the link between behavioral variation and variation in the vasopressin 1a receptor gene (*Avpr1a*) in bonobos. Chimpanzees are polymorphic for a ~360 bp deletion (DupB), which includes a microsatellite (RS3) in the 5′ promoter region of *Avpr1a*. In chimpanzees, the DupB deletion has been linked to lower sociability, lower social sensitivity, and higher anxiety. Chimpanzees and bonobos differ on these traits, leading some to believe that the absence of the DupB deletion in bonobos may be partly responsible for these differences, and to the prediction that similar associations between *Avpr1a* genotypes and personality traits should be present in bonobos. We identified bonobo personality dimensions using behavioral measures (Sociability_B_, Boldness_B_, Openness_B_, Activity_B_) and trait ratings (Assertiveness_R_, Conscientiousness_R_, Openness_R_, Agreeableness_R_, Attentiveness_R_, Extraversion_R_). In the present study we found that all 10 dimensions have nonzero heritabilities, indicating there is a genetic basis to personality, and that bonobos homozygous for shorter RS3 alleles were lower in Attentiveness_R_ and higher in Openness_B_. These results suggest that variations in *Avpr1a* genotypes explain both within and between species differences in personality traits of bonobos and chimpanzees.

There is growing evidence that personality differences are associated with fitness outcomes^1^,^2^. A remaining challenge is to identify proximate mechanisms that underlie personality variation and ultimate mechanisms that explain how this variation is maintained in populations^3^. With respect to the latter, mechanisms that have been suggested, include frequency dependent selection, mutation-selection balance, spatiotemporal variation in environmental factors, and trade-offs between alternative strategies^1^,^4^,^5^,^6^,^7^,^8^. To test these scenarios, models should incorporate explicit genetic mechanisms, since the expected response to natural selection of these traits depends on their genetic structure^9^.

To determine the proportion of personality variation attributable to genetic factors, in contrast to non-genetic factors such as the environment or error, the heritability of personality traits is typically estimated using animal models^10^. Personality traits typically have heritabilities ranging from 0 to 60% in species, such as dumpling squid (*Euprymna tasmanica*)^11^, yellow-bellied marmots (*Marmota flaviventris*)^12^, American red squirrels (*Tamiasciurus hudsonicus*)^13^, rhesus macaques (*Macaca mulatta*)^14^, chimpanzees (*Pan troglodytes*)^15^,^16^ and humans (*Homo sapiens*)^17^ (for review see^18^). Further completing the picture of how genes and environment interact to produce behavioral phenotypes is difficult, but candidate genes with large behavioral effects have been identified^19^. In humans, variation in the serotonin (*HTR2A*) and dopamine (*DRD2* & *DRD4*) receptor genes has been linked with novelty seeking^20^,^21^ and variation in the serotonin transporter gene (*5-HTT*) has been linked with anxiety^22^. In chimpanzees, variation in the tryptophan hydroxylase 2 (*TPH2*) gene is associated with neuroticism^23^ and in rhesus macaques, monoamine oxidase A (*MAOA*) gene variation is linked with aggressiveness^24^. In this study we focused on the gene coding for the vasopressin 1a receptor (*Avpr1a*). Length variations in the *Avpr1a* promoter region have been linked with behaviors related to sociability and anxiety in several mammalian species, including humans^25^.

Bonobos (*Pan paniscus*) are an interesting species in which to study associations between *Avpr1a* and personality traits as they differ from chimpanzees in the frequency of a particular microsatellite in the *Avpr1a* 5′ promoter region. Whereas bonobos have three microsatellites (RS1, RS3 and STR1), the RS3 microsatellite is often missing in chimpanzees, as it is located in a ~360 bp region (DupB) that is deleted in a majority of chimpanzees^26^,^27^. This deletion was associated with lower scores, primarily in male chimpanzees, in Sociability^28^, and also Friendliness and Smart^29^, all personality traits derived from behavioral codings of affiliative behavior and/or grooming. Associations were also found with personality dimensions derived from trait ratings. Chimpanzees with a DupB+ allele were rated higher on Conscientiousness but lower on Extraversion^16^, and DupB+ males scored higher on Conscientiousness and Dominance than females^30^. Finally, the DupB deletion was associated with a reduction in attentiveness to human social cues^31^.

Bonobos and chimpanzees are closely-related sister species^32^. We therefore expect that the presence and length variations of the RS3 microsatellite in bonobos serve similar functions in the regulation of personality traits. If so, this would support the suggestion that the DupB deletion is responsible for differences in social personality traits in these two species^26^,^33^. This study’s aim is therefore to determine the heritability of personality traits in captive bonobos^34^ and, for any heritable trait, to test whether individual differences are associated with the RS3 length polymorphism in the *Avpr1a* gene. Personality traits were measured using trait ratings^34^ and behavioral codings^35^. The trait rating approach, which relies on asking humans familiar with individual bonobos to rate them on predefined traits yielded six dimensions: Assertiveness_R_, Openness_R_, Extraversion_R_, Conscientiousness_R_, Agreeableness_R_, and Attentiveness_R_^35^. The behavioral codings approach, which relies on observing frequencies and durations of individual behaviors, identified four dimensions: Sociability_B_, Openness_B_, Boldness_B_, and Activity_B_^35^. As age and sex are important predictors for variation in personality in great apes, including bonobos^35^,^36^,^37^, we included both in our models.

## Results

### Correlations between rated and coded personality dimensions

Table 1 shows correlations between the personality dimensions derived from ratings and codings. Overall, factors were found to be largely independent. Two relatively strong and significant associations (p < 0.01) were found: a negative association between Sociability_B_ and Conscientiousness_R_ and a positive correlation between Openness_B_ and Openness_R_.

### Heritability of personality dimensions

Heritability estimates for personality dimensions using the intercept model ranged from 0.17 (Extraversion_R_) to 0.31 (Openness_R_) for rated dimensions and from 0.24 (Boldness_B_) to 0.58 (Sociability_B_) for coded dimensions (Table 2). Adding group, identity of the mother, age, sex and, in the case of coded dimensions, dominance rank to the models, led to lower heritability estimates that ranged from 0.08 (Assertiveness_R_ and Extraversion_R_) to 0.19 (Attentiveness_R_) for rated dimensions, and from 0.06 (Openness_B_) to 0.13 (Sociability_B_) for coded dimensions.

### Relationship between Avpr1a genotype and personality

We identified 11 RS3 alleles that varied in length from 463 to 489 bp (Table 3). The mean allele length of 476 bp was used as a cut-off for the classification of RS3 genotype as short or long. As the shorter alleles are less frequent, and only two individuals were homozygous for short alleles, the classification resulted in two categories: individuals that have a short allele (codings N = 16; ratings N = 31) and individuals that have two long alleles (codings N = 27; ratings N = 81).

In linear mixed models, genotype significantly predicted Openness_B_ (χ^2^(2) = 8.20 p = 0.017) and Attentiveness_R_ (χ^2^(2) = 6.02 p = 0.049). Compared to individuals with at least one short allele, individuals homozygous for long alleles scored significantly lower on Openness_B_ (β = −0.68, SE = 0.26, 95% CI = −0.82;−0.36) and higher on Attentiveness_R_ (β = 0.615, SE = 0.251, 95% CI = 0.44;0.86). No further genotype-personality associations were found: Sociability_B_ χ^2^(2) = 1.64, p = 0.44; Boldness_B_ χ^2^(2) = 4.23, p = 0.12; Activity_B_ χ^2^(2) = 1.04, p = 0.60; Assertiveness_R_ χ^2^(2) = 2.50, p = 0.29; Conscientiousness_R_ χ^2^(2) = 2.95, p = 0.23; Openness_R_ χ^2^(2) = 4.30, p = 0.12; Agreeableness_R_ χ^2^(2) = 2.29, p = 0.32; Extraversion_R_ χ^2^(2) = 2.15, p = 0.34 (see Table S1 for estimates of sex and age).

## Discussion

Personality traits showed moderate heritabilities, indicating that variation in all traits can be partly attributed to genetic variation. Moreover, individuals’ *Avpr1a* genotype was responsible for some of this variation in Attentiveness_R_ and Openness_B_.

Coded and rated personality dimensions were correlated in ways that would be expected based on the definitions of the dimensions, and the correlations were similar in size and direction to correlations described in a previous study that compared rated and coded personality factors in wild bonobos^37^. The strongest correlation was between the Openness dimensions derived from codings and ratings. However, Openness_R_ was also positively associated with Boldness_B_. This may indicate a difference in specificity between the rating method and the behavioral assessments: whereas our behavioral assays included variables related to both novelty seeking and threatening stimuli, the questionnaire was limited to items that do not necessarily distinguish between these contexts^38^. Individuals that approach novel objects and environments as well as threats could therefore possibly be rated more curious, active, and inquisitive. It is therefore likely that both methods are measuring slightly different aspects of Openness/Boldness. Conscientiousness_R_ showed a strong negative association with Sociability_B_, which is in line with the strong negative relationship between Conscientiousness_R_ and frequencies of grooming given and received, which are high in individuals that score high on Sociability_B_^35^,^36^.

Despite differences in the number of bonobos for which we had codings and ratings, the heritability estimates were comparable, and in line with estimates ranging from 0 to 0.6 found in animal personality studies^11^,^12^,^13^,^14^,^15^,^16^,^17^,^18^. Adding mother ID and group as random effects to the model significantly improved the heritability models for all of the personality dimensions except Conscientiousness_R_. Adding group and mother ID as random, and sex, age and dominance rank as fixed effects, in general attenuated the heritability estimates of all traits, meaning that our model overestimated the proportion of additive genetic variance when not taking these factors into account. The residual variance term was relatively high in most models, indicating that a large proportion of the variance in personality dimensions is due to random factors other than those included in our models. Other factors that could explain the higher similarity in bonobo personality dimensions, include rearing history^39^, rank of the mother or her presence in the group^40^, and group size^41^. However, as these measures were hard to quantify in a standardized way for the individuals included in this study, and the addition of too many random factors to the model could lead to unstable variance component estimates^42^, we chose to not incorporate them in our models.

A previous meta-analysis on genetics of personality traits in several species has indicated that different personality traits may be characterized by different heritabilities, for example, the heritability of exploratory behavior is higher than the heritability of other traits, like aggression and activity^18^. However, this does not appear to be the case for bonobos as the heritability estimates for Openness_B_ and Openness_R_ are quite low and the heritability estimates for Sociability_B_, Conscientiousness_R_ and Attentiveness_R_ are higher, although the difference is modest. Furthermore, as environmental factors and selection pressures differ for captive and wild populations, the heritability estimates for personality traits in our study cannot directly be extrapolated to the wild. This stresses the importance of studies such as this one in wild populations^10^,^11^,^13^. Nonetheless, our results indicate a genetic basis for personality in captive bonobos, and are encouraging for those who seek to identify what genetic variants are associated with personality variation.

In line with our previous study^26^, 11 *Avpr1a* alleles were found in the bonobos studied here, with a total length variation of 26 bp between the shortest and longest allele. *Avpr1a* genotype was associated with Attentiveness_R_. Bonobos with two long alleles scored higher on this trait than conspecifics with at least one short allele. Attentiveness_R_ describes high levels of intelligence, including “being highly attentive to both social and non-social cues”. As the chimpanzee Conscientiousness dimension appears to have split up into Attentiveness_R_ and Conscientiousness_R_ in bonobos, we cannot directly compare our results to previous *Avpr1a* and personality associations in chimpanzees^16^,^30^,^34^. However, as Attentiveness_R_ overlaps with the chimpanzee Conscientiousness factor, our results support the association between longer *Avpr1a* alleles and higher Conscientiousness found in chimpanzees^16^. These results potentially indicate that the association between *Avpr1a* and Conscientiousness found in chimpanzees is more driven by the item loadings that make up the bonobo Attentiveness_R_ dimension.

Furthermore, based on the description of the Attentiveness_R_ factor in bonobos, it is likely that zookeepers who frequently interact with their animals, rate bonobo Attentiveness_R_ based on their responsiveness during training and feeding sessions. In line with this, experimental testing in male chimpanzees has shown that individuals with a DupB+ allele are more responsive to socio-communicative cues of humans compared to males homozygous for the DupB deletion^31^. Bonobos are also known to outperform chimpanzees in tasks related to theory of mind^43^ and are better at gaze-following^44^. Our results indicate that variation in *Avpr1a* may be associated with within-species differences in social responsiveness, and potentially with differences between bonobos and chimpanzees. However, to conclude that this is the case requires that further evidence, using identical measures to assess social responsiveness, is collected in bonobos and chimpanzees.

The second significant association found was between RS3 genotype and Openness_B_, a personality trait derived from codings that comprised behaviors related to curiosity and exploration^35^. Although this factor was similar to Openness_R_ in its composition and was strongly and positively correlated with it, the effect was only significant for the coded factor. As the sample for the coded factor was much smaller than that for the rated factor, this may be a false positive result^45^. However, as we noted earlier, it is also possible that these personality dimensions measure somewhat different aspects of Openness or represent a different hierarchical level of the larger Openness domain. If not a false positive, then it is consistent with results from studies in rodents, where vasopressin is known to promote anxiety-like behaviors^46^,^47^ leading to a reduction in exploratory behavior^46^,^47^,^48^.

If in bonobos, as in humans, longer RS3 alleles are associated with increased transcription of *Avpr1a*^49^, this may promote anxiety related behaviors and therefore reduce levels of exploratory behavior that defines Openness_B_. In chimpanzees, a recent study found an association between the presence of DupB and an increase in anxiety-related scratching^50^. Behavioral studies further indicate that anxiety levels are higher in bonobos than in chimpanzees, as bonobos are more risk averse^51^ and neophobic in non-social contexts^52^. Anecdotal evidence also suggests that bonobos are more sensitive to captivity-induced stress than are chimpanzees^53^. Combined, these studies support our interpretation of our finding that higher frequencies of DupB+ alleles in bonobos are related to their lower Openness_B_ via increased levels of anxiety. Again, a study that uses comparable measures for anxiety and/or Openness is needed to make claims about the actual size of interspecies differences in these traits.

By providing evidence for associations between RS3 microsatellite length variations and individual variation in bonobo personality this study contributes to our knowledge of what proximate mechanisms are shaping stable individual behavioral differences in this species. As both the association with Attentiveness_R_ and Openness_B_ are consistent with previous findings on genotype associations with personality dimensions and behaviors in chimpanzees^16^,^31^,^50^, our results support our hypothesis that differences in *Avpr1a* partly explain differences in the behaviors and personalities of bonobos and chimpanzees.

## Methods

### Behavioral codings

Coding data were collected from 2012 to 2014 for 46 adolescent and adult captive bonobos (28 females and 18 males aged between 7 and 63 years old) housed in 6 European zoological parks: Planckendael in Mechelen-Belgium, Apenheul in Apeldoorn-the Netherlands, Twycross Zoo World Primate Center in the United Kingdom, Wuppertal Zoo in Germany, Frankfurt Zoo in Germany and Wilhelma Zoological and Botanical Garden in Stuttgart-Germany. All groups contained juveniles and/or infants, which were excluded from the analysis. Data were collected by NS and 8 students under her supervision. Inter-observer reliabilities reached a mean of *r* = 0.86 across all observers, and so the observations were highly reliable^54^. The methods of observation and data extraction were identical in all zoos (for details see 36).

### Data collection: naturalistic observations

Behaviors were coded using an extensive ethogram. Behavioral variables and their definitions are shown in Table S2. The total amount of data collected included 1666.15 hours of focal observations (mean 32.04 hours per individual), 10472 group scans (mean 616 per individual) and 2132 h of recording all occurrences of aggressive behavior (mean 39.5 hours per individual). Behavioral observations were coded using the Observer (Noldus version XT 10, the Netherlands).

### Data collection: experimental tests

Along with observing naturally occurring behaviors, we conducted eight group experiments, adapted from work on chimpanzees^55^. Experimental variables and their definitions are shown in Table S3. We used two predator experiments (snake and leopard), two novel food experiments (durian fruit and blue dyed pasta) and four puzzle feeder experiments (hanging barrel, barrel mesh, tubes and reel and feed). Pictures and details of behavioral measures can be found in Figs S1–S8. All experiments were captured (Canon Legria FS406, Japan) and recordings were coded using Observer Video-Pro (Noldus version XT 10, the Netherlands). Data recording started as soon as the group had access to the experiment or stimuli and was stopped after 30 minutes. All group members had access to the stimuli at the same time. In four out of six groups (Frankfurt, Twycross, Stuttgart and Apenheul), group compositions differed between behavioral tests due to artificial fission-fusion systems in these zoos. The order of the experiments was randomized and there were at least 3 days between experiments.

### Intraclass correlations and factor analysis of behavioral codings

As the definition of personality requires stability of traits between individuals across time, all variables were tested for temporal consistency and therefore data were collected in two consecutive years for each group (Table S4). Intraclass correlations were used to determine temporal stability and only variables that were stable were used to determine personality factor structure. Factor analysis revealed four factors: Sociability_B_, Boldness_B_, Openness_B_ and Activity_B_ (Table 4). Details on each item’s loading onto each dimension can be found in Table S6.

### Observer trait ratings

Trait ratings were obtained from people familiar with the bonobos, which were primarily zookeepers. These ratings were collected for 154 individuals (83 females, 71 males, age ranging 2 to 63 years), comprising about 80% of the captive population in Europe and the United States at the time ratings were collected (Table S5). Trait ratings were made on the Hominoid Personality Questionnaire (HPQ)^34^, which consists of 54 personality descriptive adjectives, each paired with a description that sets the adjective in the context of behavior. The HPQ instructs raters to make ratings on a 7-point scale (1 =  “displays total absence or negligible amounts of the trait”, 7 =  “displays extremely large amounts of the trait”). Interrater reliabilities of the adjectives were acceptable and consistency was found up to six years after the first wave of data collection^35^. Factor analysis revealed six personality factors: Assertiveness_R_, Conscientiousness_R_, Openness_R_, Attentiveness_R_, Agreeableness_R_ and Extraversion_R_ (Table 5). Details on item loadings for each factor can be found in Table S7.

### Correlations between rated and coded personality dimensions

Ratings and codings were available for 44 individuals (18 males, 26 females), enabling us to assess the Spearman rank correlations between the personality dimensions derived from coding and from ratings. Because codings were collected between 2011 and 2014, we only used ratings collected in 2012 during the second wave of data collection.

### Genotyping

We collected DNA samples for 113 subjects (62 females, 51 males, age ranging 2 to 62 years) for whom personality data were available. Behavioral coding data were available for 43 genotyped subjects (26 females, 17 males, age ranging 7 to 62 years) and trait ratings data were available for 112 genotyped subjects (61 females, 51 males, age ranging 2 to 61 years). *Avpr1a* genotyping was conducted by NS and PH in 2011–2012 and genotypes were unknown to researchers involved in the collection of codings and trait ratings to ensure that observations were not biased by knowledge of the individual genotypes. We obtained DNA from hair, tissue, or blood samples from the Centre for Research and Conservation at the Royal Zoological Society of Antwerp, Belgium (N = 54) and the institute of human genetics at the University of Freiburg (N = 5). The San Diego Zoo Institute for Conservation Research (California, United States) PCR amplified DNA that was banked in their Frozen Zoo from 54 bonobos for analysis in their genetics lab. Human DNA from the main investigators and negative control samples were included in all procedures to test for potential contamination during analysis. Approximately 20% of the samples were re-analyzed at least once to ensure correct genotyping. Studbook information was used to validate inheritance patterns of the alleles in this study.

RS3 genotyping was completed as reported in Staes *et al*.^26^ starting with an amplification of the RS3 microsatellite using a fluorescent labelled (6-FAM) forward primer: 5′-TTT TTC AGA GGG ATC CTG-3′ and reverse: 5′-GGA ATG AGT TAA CAA ATG TTG TAG-3′. Each 25 μL PCR reaction mix contained 1X QIAGEN *Taq* Buffer advanced, 200 μM dNTP’s, 1.25U 5 PRIME *Taq* DNA polymerase (5U/μL), 0.5 μM of both primers and approximately 45ng genomic DNA. PCR started with an initial incubation at 95 °C (5 min), followed by 35 cycles at 95 °C (30 s), 54 °C (40 s), 72 °C (40 s) and a final extension period of 10 min at 72 °C. Individuals were genotyped using automated capillary electrophoresis (Macrogen Inc., South Korea).

### Statistical analysis

#### Estimating heritability with MCMCglmm

To estimate heritabilities of the personality dimensions we fit linear variance component models with the MCMCglmm package in R (version 2.15.2, R Foundation for Statistical Computing, R Development Core Team 2009) 56. This function fits an animal model in a Bayesian framework. The hypothesis to be tested is that more closely-related individuals, who are more genetically similar, will have more similar personality phenotypes than more distantly-related individuals. These models can also take into account the potential role of common environmental effects that could lead individuals to be more similar to one another in their phenotype than expected. Pedigree information of the bonobo population was used to evaluate covariance between phenotypic and genetic similarity. All variance components models were run with a minimum of 1,000,000 iterations and burn-in periods of 100,000. Convergence of our model was tested using the Heidelberg stationary test, where p-values must exceed 0.05 and by using autocorrelations, which had to be smaller than 0.1 for the first lag^42^. Posterior modes of heritability estimates are reported with their credible intervals.

Sex and age were entered as fixed effects and their significance was assessed from the posterior distributions using the highest-posterior-density function (HPD interval, library coda in R)^56^. When the HPD interval did not include zero, the factor was considered significant. Age was entered as a continuous variable. For personality factors derived from behavioral observations, dominance rank was added as an additional fixed effect. Dominance rank was measured by computing normalized David’s scores for each individual^35^,^36^,^57^. David’s scores use dyadic dominance proportions to determine dominance scores, or cardinal ranks, for each individual based on the proportions of wins and losses in agonistic encounters. Winning or losing an agonistic encounter was specified by whether the individual would flee upon aggression. We then standardized the David’s scores for each group by dividing them by the number of group members. As the inclusion of fixed effects can inflate heritability estimates and reduces comparability between studies^58^, we report the basic intercept model without additional factors and the full model with only significant effects included. Zoo and identity of the mother were included as random effects in models if they decreased the model DIC value by at least two units.

#### Estimating *Avpr1a* genotype effects with linear mixed models

We estimated genotype effects using linear mixed effects models with the lmekin function of the R package coxme^59^ in R (version 3.1.0; R Core Team 2015). The lmekin function enabled us to incorporate a kinship matrix into the model, thus allowing us to correct for the effect of relatedness. Personality traits were treated as response variables and sex, genotype, and their interaction were considered as fixed effects. Genotype was entered as a classification of short versus long RS3 alleles, using the mean allele length of the population as a cutoff for the classification^49^. As only two subjects were homozygous for the short form allele, we created two genotype groups: individuals that have one or two short alleles (SS + SL) and individuals that are homozygous for two long alleles (LL). Age was entered as a fixed effects covariate and group as a random intercept. The statistical significance of genotype effects were tested in all linear mixed models only when the full model was significant as indicated by a likelihood ratio test. We assessed the significance of the full model by comparing it with a null model that lacked all fixed effects terms involving genotype. This comparison addresses multiple testing issues which otherwise would arise^60^. To test for the stability of the models we excluded groups one at a time and compared the estimates derived with those obtained from the full model. This revealed the model to be moderately stable. We inspected qq-plots and plots of residuals against fitted values to check whether the assumptions of normally distributed and homogeneous error variance were fulfilled. These did not indicate severe violations of these assumptions.

#### Ethical statement

No animals were sacrificed or sedated for the purpose of this study. All European DNA samples were provided from existing DNA databanks that collect and store samples following BIAZA guidelines that state that some material may be obtained opportunistically during health checks or other recognized husbandry procedures. Most of these samples were hair samples that were collected non-invasively. In case of blood samples, we followed the BIAZA guidelines that state that no more than 10% of samples taken for veterinary purposes can be used for secondary research purpose. Samples from San Diego Zoo animals were collected opportunistically during routine veterinary checks and approved by the SDZG IACUC (assurance# 12-023). Additional samples from other USA zoos were also collected opportunistically at AZA accredited facilities for population management purposes and are not subject to IACUC approval. Human DNA from the main investigators (NS and JMGS) was acquired non-invasively by use of buccal swabs. As the samples were collected non-invasively and only for the purpose of methodological validation, with no intent to interpret or publish further results regarding these samples, the Scientific Advisory Board of the Royal Zoological Society of Antwerp waived the requirement for human subjects approval for human tissue collection and use in this study. This research was approved by the University of Antwerp (Belgium) and endorsed by the European Breeding Program for bonobos.

## Additional Information

**How to cite this article**: Staes, N. *et al*. Bonobo personality traits are heritable and associated with vasopressin receptor gene 1a variation. *Sci. Rep.* **6**, 38193; doi: 10.1038/srep38193 (2016).

**Publisher's note:** Springer Nature remains neutral with regard to jurisdictional claims in published maps and institutional affiliations.

## Supplementary Material

Supplementary Information

## Figures and Tables

**Table 1:** Correlations between rated and coded personality dimensions.

	Assertiveness_R_	Conscientiousness_R_	Openness_R_	Attentiveness_R_	Agreeableness_R_	Extraversion_R_
Sociability_B_	0.32*	−0.50**	0.18	0.17	0.14	0.18
Openness_B_	**−**0.13	−0.30*	0.52**	**−**0.10	**−**0.10	0.33*
Boldness_B_	0.06	**−**0.04	0.37*	0.21	0.16	0.16
Activity_B_	**−**0.15	**−**0.12	0.07	0.02	**−**0.29	0.04

Spearman rank correlations. Significant effects in boldface. *significant at the 0.05 level; **significant at the 0.01.

**Table 2:** Heritability estimates for personality dimensions.

	Intercept model			Best fit model random effects						Fixed effects		
	*h*_*0*_*^2^*	95% CI	V_A_	*h^2^*	95% CI	V_A_	V_R_	V_GROUP_	V_MOTHER_	Sex	Age	Rank
Sociability_B_	0.58	(0.16, 0.94)	0.67 ± 0.72	0.13	(0.01, 0.46)	0.13 ± 0.09	0.13 ± 0.09	0.38 ± 0.35	0.07 ± 0.05	**−**0.59 ± 0.58*	/	3.29 ± 2.32*
Openness_B_	0.40	(0.05, 0.82)	0.39 ± 0.14	0.06	(0.01, 0.50)	0.07 ± 0.04	0.21 ± 0.18	0.10 ± 0.08	0.08 ± 0.06	/	**−**0.04 ± 0.02***	/
Boldness_B_	0.24	(0.06, 0.54)	0.39 ± 0.19	0.09	(0.02, 0.40)	0.12 ± 0.09	0.07 ± 0.05	0.10 ± 0.08	0.52 ± 0.49	/	/	/
Activity_B_	0.51	(0.11, 0.90)	0.49 ± 0.41	0.11	(0.02–0.63)	0.06 ± 0.04	0.36 ± 0.31	0.18 ± 0.15	0.06 ± 0.04	/	/	/
Assertiveness_R_	0.30	(0.07–0.59)	0.41 ± 0.09	0.08	(0.02–0.22)	0.05 ± 0.02	0.50 ± 0.14	0.18 ± 0.12	0.06 ± 0.02	−2.71 ± 2.6*	0.42 ± 0.24***	
Conscientiousness_R_	0.21	(0.05–0.42)	0.16 ± 0.14	0.13	(0.05–0.42)	0.13 ± 0.06	1.04 ± 0.24	/	/	/	/	
Openness_R_	0.31	(0.07–0.59)	0.41 ± 0.28	0.09	(0.03–0.27)	0.09 ± 0.05	0.67 ± 0.34	0.10 ± 0.25	0.08 ± 0.25	/	−0.05 ± 0.01***	
Attentiveness_R_	0.28	(0.06–0.54)	0.20 ± 0.11	0.19	(0.03–0.40)	0.22 ± 0.18	0.52 ± 0.20	0.17 ± 0.13	0.11 ± 0.07	/	−0.02 ± 0.01*	
Agreeableness_R_	0.25	(0.06–0.49)	0.23 ± 0.13	0.11	(0.03–0.38)	0.08 ± 0.06	0.59 ± 0.19	0.09 ± 0.07	0.06 ± 0.04	/	/	
Extraversion_R_	0.17	(0.04–0.32)	0.16 ± 0.11	0.08	(0.03–0.46)	0.08 ± 0.05	0.66 ± 0.26	0.06 ± 0.04	0.08 ± 0.06	−0.43 ± 0.31*	−0.03 ± 0.01**	

*h*_*0*_^2^ = heritability based on intercept model. *h*^2^ *= *heritability corrected for environmental factors. V_A_ =  additive genetic variance, V_R_ =  residual variance, V_GROUP_ =  group variance, V_MOTHER_ =  variance explained by mother. For Sex the effect size is given for males with significance level. The effect of Rank was only tested for dimensions derived from codings. *Significant at the 0.05 level **Significant at the 0.01 level. ***Significant at the 0.001 level.

**Table 3:** RS3 allele frequencies in two bonobo populations for whom personality traits were assessed.

Allele (bp)	Ratings		Codings	
	Frequency	Percentage (%)	Frequency	Percentage (%)
463	7	0.03	3	0.03
465	25	0.11	14	0.16
469	2	0.01	0	0.00
477	10	0.04	5	0.06
479	26	0.12	9	0.10
481	37	0.16	12	0.14
483	14	0.06	10	0.12
484	40	0.18	18	0.21
485	54	0.24	13	0.15
487	1	0.00	1	0.01
489	10	0.04	1	0.01

**Table 4:** The behavioral contents of the coded personality dimensions.

Factor	Adjectives loading on to factor
**Sociability**_**B**_	*+ Grooming frequencies + Grooming density + Neighbors + Grooming diversity − Latency to approach puzzles/durian − autogroom*
**Openness**_**B**_	*+ Approaches to puzzles/others + Play + Proximity to puzzles + Taste pasta*
**Boldness**_**B**_	*+ Approaches to leopard + displays to leopard + proximity to leopard + Aggression received*
**Activity**_**B**_	*+ Self-scratching − Activity*

**Table 5:** The adjectival contents of the rated personality dimensions.

Factor	Adjectives loading on to factor
Assertiveness_R_	*+ Independent + Dominant + Cool + Stable + Decisive + Persistent − Excitable − Dependent − Submissive− Vulnerable − Fearful − Timid − Anxious*
Conscientiousness_R_	*+ Gentle + Predictable − Impulsive − Manipulative − Reckless − Defiant − Erratic − Jealous − Irritable − Stingy − Aggressive − Bullying*
Openness_R_	*+ Active + Playful + Inquisitive + Inventive + Imitative + Innovative + Curious − Lazy − Conventional*
Attentiveness_R_	*+ Intelligent − Unperceptive − Distractable − Thoughtless − Clumsy − Disorganized*
Agreeableness_R_	*+ Friendly + Affectionate + Protective + Sympathetic + Helpful + Sociable + Sensitive*
Extraversion_R_	*− Individualistic − Autistic − Depressed − Solitary*
