# Supplementary material for: Bonobo personality traits are heritable and associated with vasopressin receptor gene 1a variation
Source: Sci Rep. 2016 Dec 2;6:38193. doi: 10.1038/srep38193 (PMC5133571; doi:10.1038/srep38193)
Supplement: Supplementary Information [file srep38193-s1.pdf]

Bonobo personality traits are heritable and associated with vasopressin receptor gene 1a variation

Nicky Staes [(1,2,3)*], Alex Weiss [(4,5)], Philippe Helsen [(1,2)], Marisa Korody [(6)], Marcel Eens [(2)] & Jeroen M.G. Stevens [(1,2)]

*corresponding author: *nstaes@gwu.edu*

[1] *Centre for Research and Conservation, Royal Zoological Society of Antwerp, Antwerp, Belgium*

[2] *Behavioural Ecology & Ecophysiology Group, Department of Biology, University of Antwerp, Antwerp, Belgium*

[3] *Center for Advanced Study of Human Paleobiology, Department of Anthropology, George Washington University, Washington DC, United States of America*

[4] *Department of Psychology, School of Philosophy, Psychology and Language Sciences, The University of Edinburgh, United Kingdom*

[5] *The Scottish Primate Research Group, United Kingdom*

[6] *San Diego Zoo Institute for Conservation Research, California, United States of America*

Supplementary information

Table S1 : Linear mixed model output model for personality dimensions with group as random intercept

	Response	Main effects	est	SE	min	max
Ratings	$Assertiveness_R$	Sex Male	-1,035*	1,157	-1,698	-0,469
		Genotype Long/Long	0,318	0,316	0,133	0,449
		Age	0,080	0,091	0,030	0,121
	$Conscientiousness_R$	Sex Male	-1,582	1,196	-2,110	-0,925
		Genotype Long/Long	-0,538	0,322	-0,707	-0,377
		Age	0,072	0,094	-0,001	0,108
	$Openness_R$	Sex Male	0,034	0,848	-0,205	0,477
		Genotype Long/Long	0,363	0,233	0,255	0,532
		Age	-0,630*	0,067	-0,702	-0,599
	$Agreeableness_R$	Sex Male	-0,751	0,951	-1,290	-0,294
		Genotype Long/Long	0,147	0,263	-0,072	0,286
		Age	0,094	0,075	0,047	0,147
	$Attentiveness_R$	Sex Male	1,119	0,914	0,748	1,892
		Genotype Long/Long	0,615*	0,251	0,443	0,857
		Age	-0,038	0,072	-0,119	0,015
	$Extraversion_R$	Sex Male	-1,670*	1,001	-2,463	-0,870
		Genotype Long/Long	-0,034	0,273	-0,211	0,067
		Age	-0,222*	0,079	-0,301	-0,189
Codings	$Sociability_B$	Sex Male	0,724*	1,292	0,111	1,893
		Genotype Long/Long	0,344	0,353	-0,001	0,557
		Age	0,027	0,127	-0,041	0,133
	$Openness_B$	Sex Male	-0,975*	0,968	-1,205	-0,187
		Genotype Long/Long	-0,677	0,259	-0,815	-0,357
		Age	-0,574*	0,093	-0,654	-0,509
	$Boldness_B$	Sex Male	0,081	1,336	-1,011	1,625
		Genotype Long/Long	0,617	0,351	0,263	0,717
		Age	-0,281	0,127	-0,563	-0,101
	$Activity_B$	Sex Male	0,871*	1,097	0,030	1,381
		Genotype Long/Long	0,277	0,298	-0,347	0,663
		Age	-0,151	0,107	-0,246	-0,040

* indicates p<0.05

Table S2: Behavioral variables used to determine personality model based on codings

Variable	Definition	Calculated as
**Activity**	Time spent not resting, sleeping, sitting or auto-grooming	Focal observation time minus time spent resting, sleeping, sitting or autogrooming, divided by total focal observation time
**Submission**	Frequency of submissive behaviors	Frequency per hour of flee, flinch and crouch behaviors during focal observations of all subjects in the group
**Aggression given**	Frequency of performed aggressive behaviors	Frequency per hour of aggressive intentions, long charges, short charges, direct displays, mutual displays and parallel displays with victim showing grin, flee or counter aggression during focal observations of all subjects in the group
**Aggression received**	Frequency of received aggressive behaviors	Frequency per hour of received aggressive intentions, long charges, short charges, direct displays, mutual displays and parallel displays during focal observations of all subjects in the group
**Number of neighbors**	Average number of group members in proximity to subject	Average number of group members in subject's proximity in scans recorded as 'sit with' (S)
**Approach others**	Frequency of focal subject approaching others	Frequency per hour of focal subject approaching and staying in 2 m proximity of others
**Being approached**	Frequency of focal subject being approached by others	Frequency per hour of focal subject being approached
**Grooming density given**	Number of group members groomed by subject	Number of individuals the subject gives grooming to divided by total available grooming partners
**Grooming density received**	Number of group members that groom the subject	Number of individuals the subject receives grooming from divided by total available grooming partners
**Grooming diversity index**	Equality of grooming effort given to different grooming partners	Shannon-Wiener diversity index corrected for group size effect (see text for formula)
**Grooming given**	Time spent grooming others	Time spent grooming divided by focal observation time
**Grooming received**	Time spent being groomed by others	Time spent being groomed divided by focal observation time
**Play**	Time spent playing with group members	Total duration of calm play and rough play divided by focal observation time
**Scratching**	Time spent self-scratching. both gentle and rough	Total duration of rough and gentle auto-scratching behaviors divided by focal observation time
**Auto-grooming**	Time spent self-grooming	Total duration of auto-grooming divided by focal observation time
**Socio-sexual behaviors**	Frequency of short duration affinitive behaviors	Frequency/h of point affinitive behavior by focal subject (affiliative touch, embrace, seks inspect, seks present, copulation, non-copulary mount, oral genital massage, genital massage)

Table S3: Variables, their definitions and the experiments they were sampled in using the experimental setup

Variable	Definition	Calculated as	**Type of experiment**
**Manipulate Puzzle**	Percentage of time spent manipulating the puzzle	Duration of working the puzzle while touching it divided by total duration of the experiment	Hanging barrel<br>Crate with mesh<br>Reel and feed<br>Turning tube
**Tool use**	Percentage of time spent manipulating the puzzle with tools	Duration of tool use divided by total duration of the experiment	Crate with mesh
**Latency to approach**	Latency to first approach the experiment object within arm's reach of it	Duration of interval between entering of the group in the enclosure and first approach towards experiment object	All experiments
**Number of approaches**	Number of approaches made towards the experiment object throughout the duration of the experiment	Absolute count of approaches made during the total duration of the experiment	Hanging barrel<br>Crate with mesh<br>Reel and feed<br>Turning tube<br>Leopard
**Proximity**	Time spent within 2m proximity to the experiment object while not touching it	Summed durations of time spent within 2m of the object divided by total duration of the experiment duration	Hanging barrel<br>Crate with mesh<br>Reel and feed<br>Twisting tube<br>Leopard
**Taste novel food**	Tasting a novel food item	One zero sampling of whether they try the novel food during the total duration of the experiment	Durian<br>Blue pasta
**Poke leopard**	Using a tool to poke at the fake leopard	Absolute count of times the subject tries to poke at the fake predator with a tool during the total duration of the experiment	Leopard
**Knock barrier**	Making forceful contact with the barrier separating the subject from the fake leopard	Absolute count of times the subject makes forceful contact with barrier during the total duration of the experiment	Leopard. snake

Table S4: Group composition, time of behavioral data collection and observers

	Zoo	Adult and subadult	Juveniles	Period	Observers
**Round 1**	PL	3M / 2F	1M / 1F	Nov. 2011 - Jan. 2012	AP
	AP	2M / 4F	2M / 2F	Mar. - Apr. 2012	AS, NS
	WI	2M / 5F	1F	Feb. - Mar. 2012	AP
	FR	3M / 6F	3M / 2F	Apr. - May 2012	SR, NS
	WU	3M / 3F	3M	May. - Jun. 2012	SR, NS
	TW	3M / 6F	1M / 2F	Sep. – Nov. 2012	NS
**Round 2**	PL	3M / 3F	1M / 1F	Nov. 2012 - Jan. 2013	WR
	AP	2M / 4F	1M / 2F	Feb. - May 2013	LJ
	WI	2M / 4F	2F	Nov. - Dec. 2012	NS
	FR	3M / 7F	3M / 3F	Feb. - Apr. 2014	MW
	WU	3M / 2F	2M	Jan. - Mar. 2013	WR
	TW*	3M / 5F	1M / 2F	Aug. – Oct. 2013	MB
**Round 3**	WI	3M / 9F	2M / 2F	May – Jul. 2013.	MB

PL = Planckendael, AP=Apenheul, WI=Wilhelma Zoological and Botanical Garden, FR=Frankfurt Zoo, WU=Wuppertal Zoo, TW=Twycross Zoo World Primate Center. * In Twycross collection of natural observations but no experimental data was done in round 2

Table S5: Group composition, time of behavioral data collection and observers

	Zoo	Adult and subadult	Juveniles	Period of rating	Raters
**Round 1**	AP	2M / 5F	2F	2006	RB, JR
	CI	3M / 2F	2M	2007	UN
	CO	5M / 4F	/	2006	AG
	FR	1M/ 6F	2M / 1F	2007	CK
	KO	3M / 3F	/	2007	ME, UN
	LE	3M / 2F	/	2007	FM
	ME	1M/ 3F	/	2006	RS
	PL	3M / 3F	/	2006-2007	MR, BJ, AM, JJ
	WI	5M / 4F	/	2007	RS, KR, BJ, K, SP, SC
	TW	3M / 4F	1M / 1F	2006	CH
	GA	5M / 2F	/	2008	KA, MA, TA
	WU	4M / 2F	/	2007	SQ, PO, ST
**Round 2**	AP	2M / 4F	2M / 1F	2012	IS, JR, DJ,
	CO	6M / 6F	2M / 2F	2012	AG, KB, JL
	FO	3M / 3F	1F	2012	AV, EH, DA
	FR	3M / 6F	3M / 2F	2012	BL, CK, CO, JA, RB, LA, VO
	JA	3M / 5F	1F	2012	SB, SZ
	MI	5M / 8F	1M / 2F	2012	BB
	PL	3M / 3F	1M / 1F	2012	AM, AP, ED, JJ, LP
	SD	2M / 4F	/	2012	DG, PS
	WI	3M / 7F	2F	2012	AP, KI, SP
	TW	3M / 6F	1M / 1F	2012	DS, EC, MC

AP=Apenheul, CI=Cincinnati, CO=Columbus, FR=Frankfurt, KO=Koln, LE=Leipzig, ME=Memphis, PL=Planckendael, WI=Wilhema Zoo, TW=Twycross, GA=GATI, WU=Wuppertal, FO=Fortworth, JA=Jacksonville, MI=Milwaukee, SD=San Diego Wild Animal Park

Table S6: Variable loadings dimension reduction model derived from codings

Table: Varimax rotated Factor Loadings

	Factor				
Variable	Sociability	Openness	Boldness	Activity	h^2
Grooming Received	**0.83**	-0.03	-0.03	-0.20	0.74
Grooming Density Received	**0.76**	-0.07	-0.12	-0.14	0.68
Nr of Neighbours	**0.71**	0.13	0.13	0.04	0.54
Grooming Given	**0.67**	0.18	0.13	-0.39	0.69
Latency to Approach Puzzle	**-0.66**	-0.49	0.02	-0.24	0.79
Grooming Density Given	**0.64**	0.20	0.33	-0.42	0.84
Latency to Approach Durian	**-0.64**	-0.23	-0.01	-0.14	0.47
Grooming Diversity Index	**0.53**	0.12	0.19	-0.36	0.67
Autogroom	**-0.48**	0.10	-0.39	-0.01	0.46
Puzzle Nr of Approaches	0.08	**0.91**	0.13	-0.06	0.83
Play	-0.07	**0.70**	0.00	-0.22	0.63
Time in Proximity to Puzzle	0.20	**0.68**	-0.31	-0.03	0.59
Approach others	0.05	**0.65**	0.35	-0.27	0.69
Taste Pasta	0.27	**0.41**	0.20	-0.11	0.42
Leopard Nr of Approaches	0.02	0.11	**0.82**	-0.02	0.67
Leopard Nr of Displays	0.21	0.07	**0.62**	0.01	0.48
Time in Proximity to Leopard	0.10	-0.08	**0.59**	0.44	0.54
Aggression Received	-0.37	0.12	**0.54**	-0.31	0.54
Scratch	-0.10	-0.17	0.19	**0.69**	0.66
Activity	0.29	0.30	0.26	**-0.53**	0.65
Eigenvalue	5.98	2.85	2.59	1.73	
% variance explained	29.92	14.25	12.93	8.65	

Table S7: Item loadings dimension reduction model derived from ratings

	Factor						
Adjective	Ass_R	Con_R	Ope_R	Att_R	Agr_R	Ext_R	h2
Anxious	**-0.81**	-0.04	-0.16	-0.07	-0.03	-0.17	0.71
Timid	**-0.76**	0.27	-0.12	-0.07	-0.05	-0.12	0.69
Fearful	**-0.75**	0.06	-0.17	-0.16	-0.10	-0.12	0.64
Independent	**0.71**	-0.20	-0.02	0.13	0.04	-0.30	0.66
Dominant	**0.68**	-0.52	-0.26	0.07	0.11	0.07	0.82
Vulnerable	**-0.66**	0.22	0.10	0.00	-0.19	-0.37	0.67
Submissive	**-0.64**	0.43	0.18	-0.08	0.04	-0.17	0.66
Cool	**0.63**	0.13	-0.04	0.08	0.26	-0.05	0.50
Stable	**0.62**	0.24	0.13	0.11	0.20	0.03	0.52
Dependent	**0-.61**	0.12	0.17	-0.37	0.20	0.35	0.71
Decisive	**0.54**	-0.07	0.09	0.40	0.15	0.00	0.49
Persistent	**0.52**	-0.40	0.15	0.14	0.13	0.05	0.49
Excitable	**-0.52**	-0.30	-0.05	-0.12	0.00	-0.01	0.39
Cautious	-0.36	0.31	-0.10	0.20	0.35	-0.08	0.41
Quitting	-0.20	0.08	-0.19	-0.20	0.12	0.00	0.14
Bullying	0.27	**-0.76**	0.01	0.08	-0.16	0.06	0.68
Aggressive	0.15	**-0.75**	0.10	0.08	-0.14	0.05	0.63
Stingy	0.20	**-0.72**	0.02	-0.14	-0.03	-0.05	0.58
Irritable	-0.12	**-0.70**	-0.17	-0.09	-0.14	-0.21	0.60
Jealous	-0.04	**-0.68**	0.18	-0.15	-0.07	-0.08	0.53
Gentle	-0.02	**0.67**	-0.02	0.04	0.53	-0.06	0.73
Erratic	-0.29	**-0.60**	-0.02	-0.29	-0.06	-0.18	0.57
Defiant	0.11	**-0.59**	0.23	-0.11	-0.19	-0.05	0.46
Reckless	0.07	**-0.58**	0.19	-0.38	-0.21	-0.05	0.57
Manipulative	0.21	**-0.55**	0.05	0.17	0.14	0.14	0.42
Impulsive	-0.29	**-0.48**	0.28	-0.15	-0.14	-0.06	0.45
Predictable	0.21	**0.41**	-0.23	0.00	0.26	-0.05	0.33
Active	-0.13	-0.12	**0.77**	-0.08	0.09	0.21	0.68
Playful	-0.08	-0.05	**0.73**	-0.04	0.10	0.20	0.59
Inquisitive	0.22	-0.01	**0.68**	0.21	0.11	0.03	0.57
Inventive	0.34	-0.05	**0.64**	0.20	0.03	-0.24	0.62
Imitative	-0.08	0.04	**0.63**	-0.22	0.17	0.13	0.50
Innovative	0.33	0.01	**0.63**	0.07	0.05	-0.21	0.56
Conventional	-0.08	0.16	**-0.61**	0.09	0.31	-0.01	0.51
Curious	0.15	-0.14	**0.58**	0.23	0.17	-0.07	0.46
Lazy	0.12	0.03	**-0.54**	-0.37	0.06	-0.30	0.54
Unemotional	0.15	0.15	-0.25	-0.11	0.12	-0.01	0.14
Disorganized	-0.18	-0.15	-0.03	**-0.71**	-0.16	-0.20	0.63
Intelligent	0.18	-0.07	0.15	**0.68**	0.34	0.06	0.65

Clumsy	0.04	0.00	-0.04	**-0.68**	-0.06	-0.15	0.50
Thoughtless	-0.18	-0.17	-0.09	**-0.68**	-0.18	-0.03	0.56
Distractible	-0.22	-0.11	0.06	**-0.59**	-0.05	0.12	0.42
Unperceptive	-0.33	-0.15	-0.16	**-0.50**	0.09	-0.24	0.47
Friendly	-0.01	0.40	0.18	-0.12	**0.71**	0.24	0.77
Affectionate	0.21	0.28	0.18	0.08	**0.71**	0.32	0.76
Protective	0.30	-0.04	-0.13	0.27	**0.70**	-0.02	0.66
Sympathetic	0.07	0.36	0.00	0.17	**0.62**	-0.13	0.56
Helpful	0.07	0.15	0.17	0.31	**0.59**	0.04	0.51
Sociable	0.15	-0.06	0.34	-0.01	**0.56**	0.34	0.57
Sensitive	0.25	0.15	-0.15	0.48	**0.53**	-0.03	0.62
Solitary	-0.17	0.17	-0.26	-0.09	-0.14	**-0.67**	0.61
Depressed	-0.42	-0.13	-0.18	-0.21	-0.07	**-0.67**	0.72
Autistic	-0.43	-0.23	-0.08	-0.34	-0.06	**-0.43**	0.55
Individualistic	0.14	-0.19	0.23	-0.18	-0.04	**-0.43**	0.33
Proportion of variance	0.14	0.12	0.09	0.08	0.08	0.05	

Note. aFactor was reflected. Ast = Assertiveness. Con = Conscientiousness. Opn = Openness. Att = Attentiveness. Agr = Agreeableness. Ext = Extraversion. Loadings ≥ |0.4| in boldface.

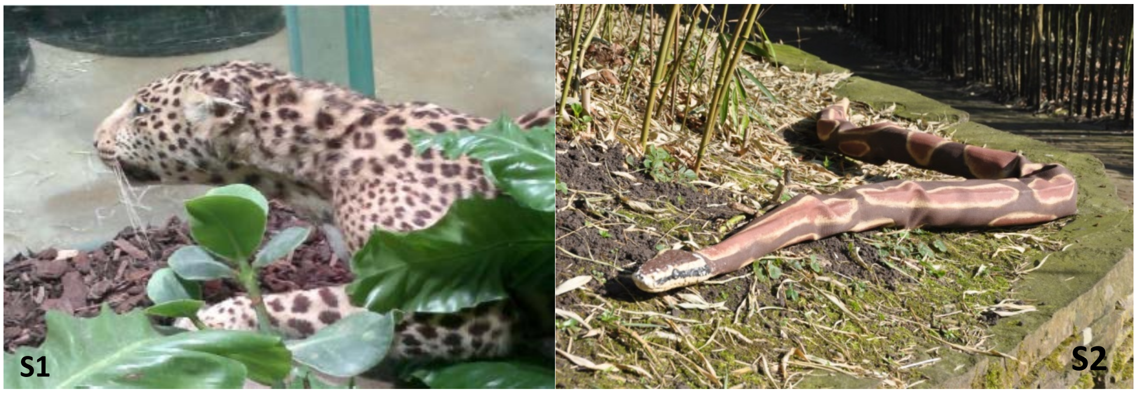

Fig S1-S2: **Predator experiments.** In the predator experiments, two model predators were used: a taxidermied leopard (S1) in crouching position with bared teeth and a 4 meter snake (S2) that was made out of a fire hose and clay and painted to resemble a python. Both predator models were placed in sight, but out of reach of the bonobos. The bonobos' behavior towards the models was scored: number of approaches, time spent in proximity and number of displays against the mesh, including both poking with sticks and banging the mesh, behind which the predator was placed. The snake experiment was dropped from further analysis as the response of the bonobos towards the model was very modest and little variation among individuals was measured

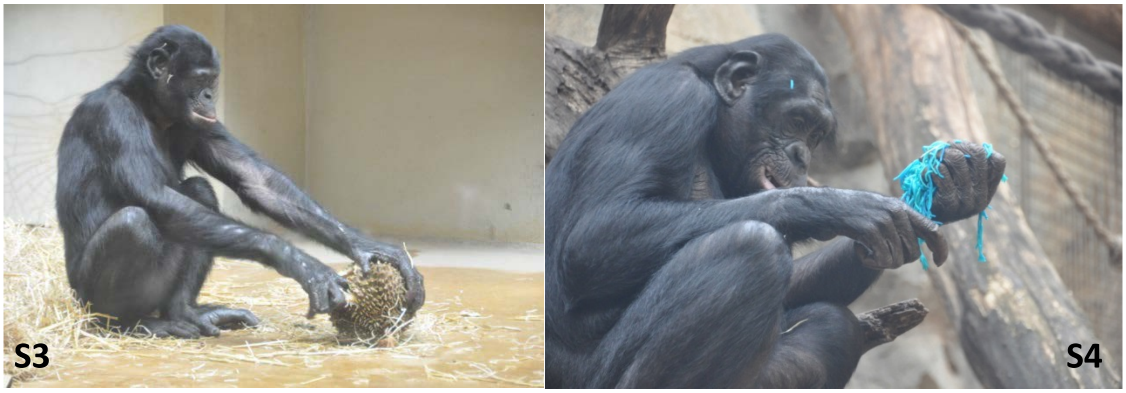

Fig S3-S4: Novel food experiments. In the novel food experiments, bonobos were given durian fruit (S3) and pasta that was dyed blue with an edible food dye (S4). During the durian experiment, two whole durian fruits were placed in the enclosure a few meters apart to avoid monopolization by one individual. Two cameras were used to allow tracking both fruits at the same time. During the pasta experiment, 250 grams of pasta were boiled, left to cool and then put in two piles in the enclosure, some meters apart, again to avoid monopolization. In these novel food experiments we measured latency to approach the novel food closer than 2 meters (in seconds) and whether they tasted it (one/zero). When an individual did not approach the food item, it was given the maximum duration of 1800 s.

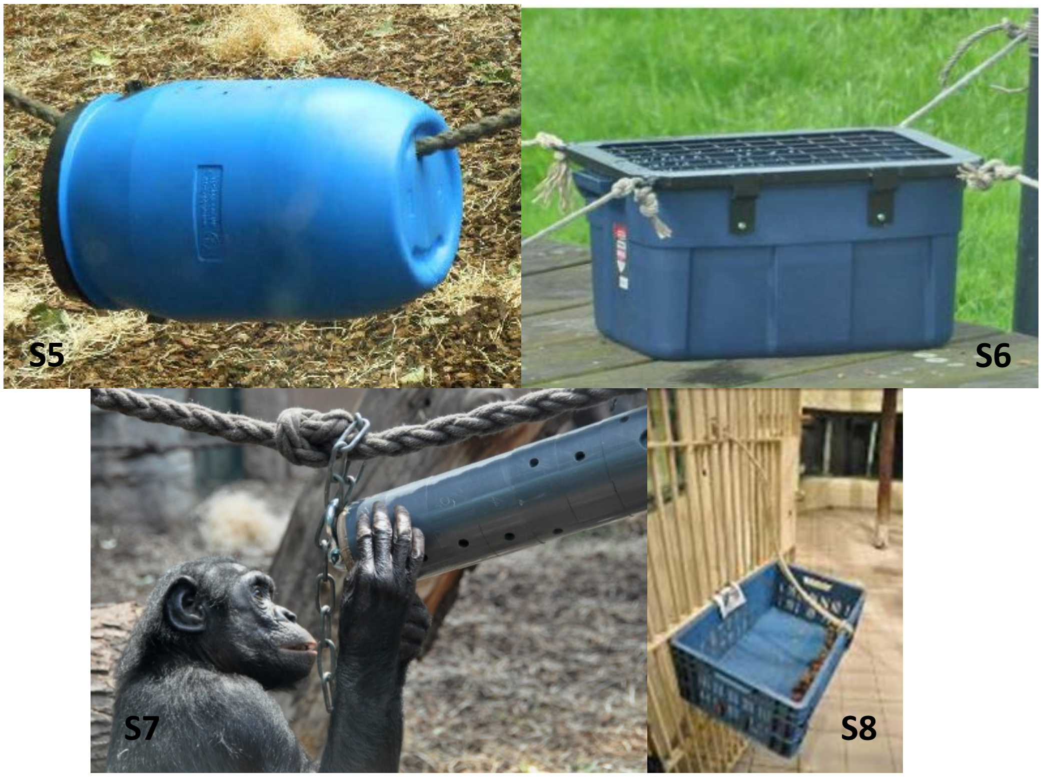

Fig S5-S8: Puzzle feeder experiments. In each of the four puzzle feeder experiments, bonobos were given a different foraging device. The first was a hanging barrel filled with seeds (“Hanging Barrel”) (S5). The second was a barrel filled with water and pieces of pear that sink to the bottom with a squared mesh on top too small to fit an adult bonobo’s hand through (“Barrel Mesh”) (S6). The third was a seed-filled hanging double tube system that has to be rotated in order to release its content (“Tubes”)(S7). The final one was a crate hanging from the outside of a mesh door next (“Reel and feed”)(S8) to the enclosure that has to be tilted using a rope that hangs in the enclosure. This way the bonobos were able to obtain fruit and/or vegetables in the crate that were part of the daily diet. For all puzzle feeders the following variables were measured: a) the time in seconds to approach the puzzle within 2 meters; b) the proportion of time spent manipulating the puzzle; c) the proportion of time spent within 2 meters of the puzzle without touching it; d) the number of times they approached the puzzle. As sticks were needed to get to the pear in the “Barrel with Mesh” condition, we measured the proportion of time during which subjects used tools.

.
